# Conserved cellular patterning in the mesophyll of rice leaves

**DOI:** 10.1002/pld3.549

**Published:** 2023-12-04

**Authors:** Jen Sloan, Saranrat Wang, Qi Yang Ngai, Yi Xiao, Jodie Armand, Matthew J. Wilson, Xin‐Guang Zhu, Andrew J. Fleming

**Affiliations:** ^1^ Plants, Photosynthesis and Soil, School of Biosciences University of Sheffield Sheffield UK; ^2^ Carl R. Woese Institute for Genomic Biology University of Illinois at Urbana Champaign Urbana IL USA; ^3^ Institute of Plant Physiology & Ecology, Shanghai Institutes for Biological Sciences Chinese Academy of Sciences Shanghai China

**Keywords:** cell shape, cell size, light, mesophyll, photosynthesis, rice

## Abstract

The mesophyll cells of grass leaves, such as rice, are traditionally viewed as displaying a relatively uniform pattern, in contrast to the clear distinctions of palisade and spongy layers in typical eudicot leaves. This quantitative analysis of mesophyll cell size and shape in rice leaves reveals that there is an inherent pattern in which cells in the middle layer of the mesophyll are larger and less circular and have a distinct orientation of their long axis compared to mesophyll cells in other layers. Moreover, this pattern was observed in a range of rice cultivars and species. The significance of this pattern with relation to potential photosynthetic function and the implication of the widespread use of middle layer mesophyll cells as typical of the rice leaf have been investigated and discussed.

## 1 INTRODUCTION

Sandwiched between the upper and lower surfaces of the leaf lies the mesophyll tissue, the main site of photosynthesis in angiosperms. The structure of the mesophyll is important since it directly influences a number of parameters related to leaf function, most notably carbon assimilation (Lundgren & Fleming, 2020; Terashima et al., 2011). For example, to reach a chloroplast for photosynthetic fixation, CO_2_ must diffuse into the leaf via the stomata, through the intercellular airspace and into the mesophyll cells. The area of mesophyll cell wall exposed to intercellular air space (*S*_*mes*_) and the relative proportions of air, cell, and cell wall in the mesophyll can determine its resistance to CO_2_ diffusion (Evans, 2021; Ren et al., 2019), which when high can limit photosynthesis (Flexas et al., 2012). These parameters will be determined by the size, shape, and pattern of the mesophyll cells (Baillie & Fleming, 2020; Théroux‐Rancourt et al., 2021). In a similar way, mesophyll structure also influences the rate of water loss during transpiration, as CO_2_ and water travel in opposite directions along a common pathway through the mesophyll (Baillie & Fleming, 2020). Light absorption inside the leaf is likewise affected by the size, shape, and distribution of mesophyll cells, with elongated palisade cells observed in eudicots facilitating the penetration of light deeper into the leaf and the more irregular shape of the spongy mesophyll cells helping to scatter light and maximize absorption (Gotoh et al., 2018; Holloway‐Phillips, 2019; Terashima et al., 2016). Although the lignified vasculature plays a major role in providing a scaffold (Sack & Scoffoni, 2013) that allows the light absorbing mesophyll slung between the vascular tissue to be oriented for light capture, the structure of the mesophyll will also play a role in leaf mechanics. Finally, the mesophyll functions to generate carbon‐rich metabolites, which are exported to the rest of the plant via the vasculature; thus, it is possible that the number, size, and distribution of the intervening mesophyll cells might influence the ease of metabolite transport into and out of the leaf.

Clearly, leaf structure/function relationships are complex and challenging to understand. An extensive literature exists on the topic, using a range of descriptive, correlative, and functional approaches, which have led to key insights (Giuliani et al., 2013; Lehmeier et al., 2017; Mathan et al., 2021; Sage & Sage, 2009; Terashima et al., 2011; Wilson et al., 2021). With the advent of increasing computational power, modeling approaches have been used to address fundamental questions of how leaf structure relates to function (Aalto & Juurola, 2002; Earles et al., 2018; Gago et al., 2020; Théroux‐Rancourt et al., 2021; Xiao et al., 2016; Xiao & Zhu, 2017). For example, in recent work from the Fleming group, a computational model of rice leaf photosynthesis, *eLeaf* was developed, which incorporated a 3D structure of IR64 rice leaves abstracted from image data of rice leaves at various resolutions using a range of approaches (Xiao et al., 2023). The model utilized an idealized version of these data, which was sufficient for the model to successfully capture the measured photosynthetic performance of rice leaves. Nevertheless, it was clear from visual inspection that the modeled rice leaf architecture was distant in term of variation in cell size and shape from that observed in histological sections. To start to address this issue, a quantitative analysis of the mesophyll was performed in both IR64 and a range of other rice cultivars and species. The results, reported here, indicate that the rice leaf mesophyll had a more complex and ordered cellular architecture than previously acknowledged. A 2D modeling approach was then utilized, and the significance of this particular mesophyll structure to the photosynthetic performance of the rice leaf was explored.

## 2 MATERIALS AND METHODS

### 2.1 Plant material and growth conditions

*Oryza sativa* (IR64) seeds were kindly gifted to us by Professor Julie Gray. *O. sativa* (Indica) MR220, *O. sativa* (fragrant) MRQ76, and *O. sativa* (Indica) Malinja were provided by the Malaysian Agricultural Research and Development Institute, Kuala Lumpur, Malaysia. *Oryza punctata*, *Oryza meridionalis*, and *Oryza latifolia* were provided by the International Rice Research Institute, Los Baños, Philippines. Rice plants were grown in a Conviron controlled environment chamber at 70% relative humidity, in a 12 h/12 h light/dark cycle at 28°C/24 °C with a light intensity of 750 μmol m^−2^ s^−1^ at canopy height. Plants were germinated on filter paper with 15 mL water in petri dishes and then transferred to 13D pots (0.88 L) filled with 71% Kettering Loam (Boughton, UK), 23.5% Vitax John Innes No. 3 (Leicester, UK), 5% silica sand and 0.5% Osmocote Extract Standard 5–6 month slow‐release fertilizer (ICL, Ipswich, UK) by volume, saturated with water. Plants were grown for 4 to 5 weeks before leaf samples were collected for imaging.

### 2.2 Microscopy

All samples were taken from the middle 3 cm portion of the fully expanded 6th leaf. IR64 plants were harvested 21 days after sowing. All other plants were harvested 28 days after sowing.

**FIGURE 1:**
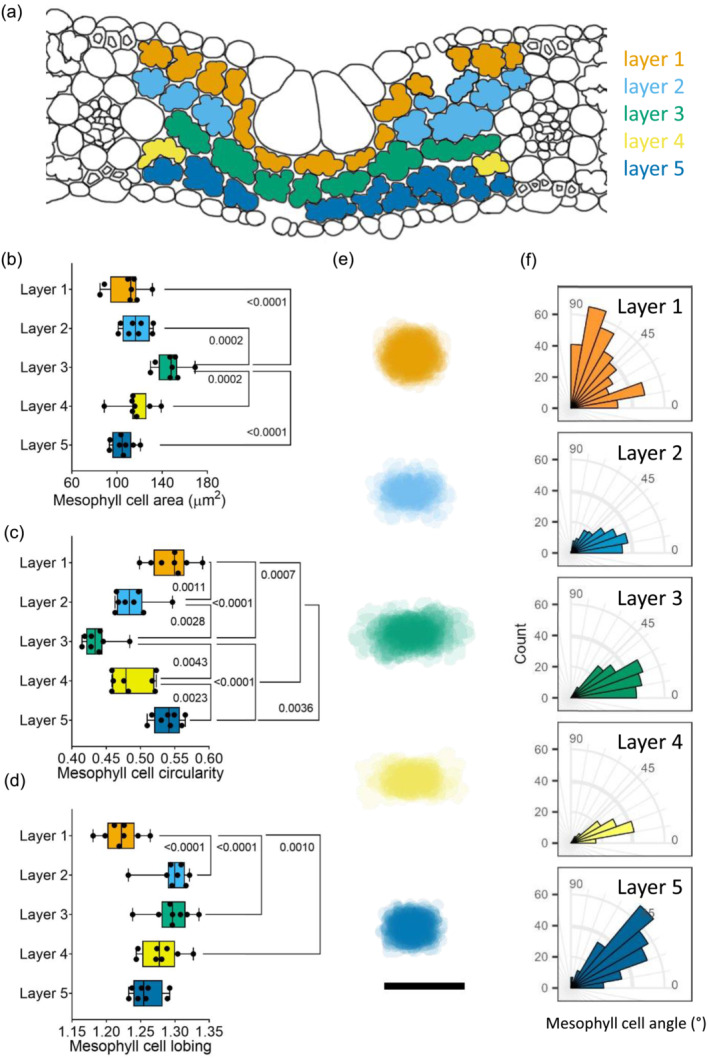
Cell layers in the mesophyll of rice IR64 can be distinguished by size, shape, and orientation. (a) Representation of rice mesophyll with different cell layers highlighted from Layer 1 (touching the adaxial epidermis) to Layer 5 (touching the abaxial epidermis). Layer 3 is a continuous row of cells between the two minor veins. (b–d) Measurement by layer of IR64 mesophyll cell area (b), circularity (c), and lobing (d). Whiskers show min–max; average line represents the mean. For (b–d), one‐way ANOVA (*p* < .0001) followed by Tukey's multiple comparison test revealed differences between cell layers, with *p* values as shown (*n* = 8). (e) Mesophyll cell projections of all cells in each layer from one representative individual. Scale bar = 20 μm. (f) Angle of long axis of mesophyll cells.

**FIGURE 2:**
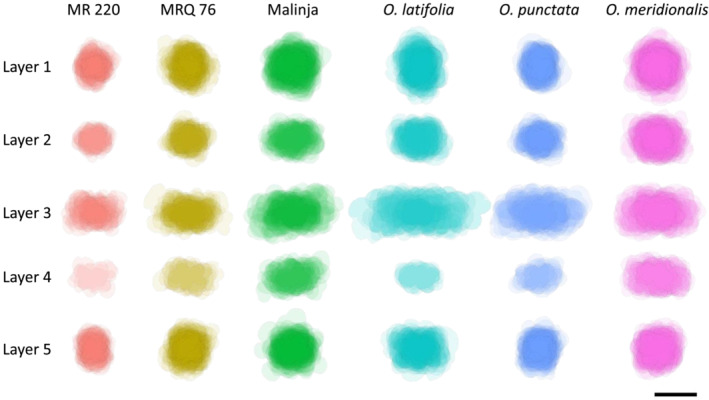
Mesophyll cell projections capture the pattern of cell size, shape, and orientation across leaf layers in a range of rice varieties. Mesophyll cell projections of all cells in each layer from one representative individual for each rice variety. Scale bar = 20 μm.

**FIGURE 3:**
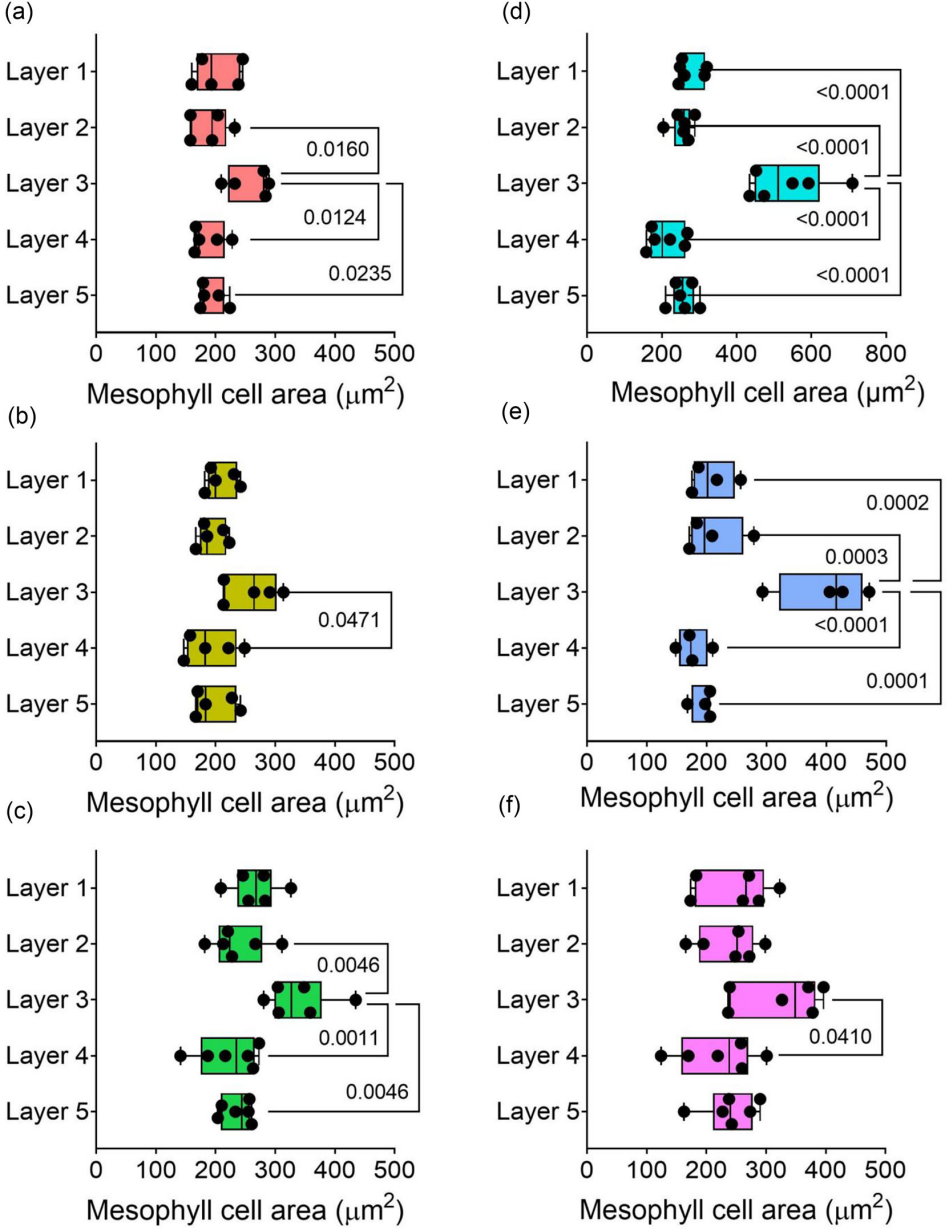
Layer 3 mesophyll cells are the largest in a range of rice varieties. (a) *O. sativa* MR220, (b) *O. sativa* MRQ76, (c) *O. sativa* Malinja, (d) *O. latifolia* , (e) *O. punctata* , (f) *O. meridionalis* . Note the different x axis scale in panel (d). Whiskers show min–max; average line represents the mean. One‐way ANOVA revealed a difference in individual cell area by layer in all lines: (a) *p* = .0081, *n* = 6; (b) *p* < .0001, *n* = 6; (c) *p* = .0368, *n* = 5; (d) *p* < .0001, *n* = 4; (e) *p* = .0009, *n* = 6; (f) *p* = .467, *n* = 6. Multiple pairwise comparisons (Tukey) are shown, with *p* values when lower than .05 (*n* = 4–6).

**FIGURE 4:**
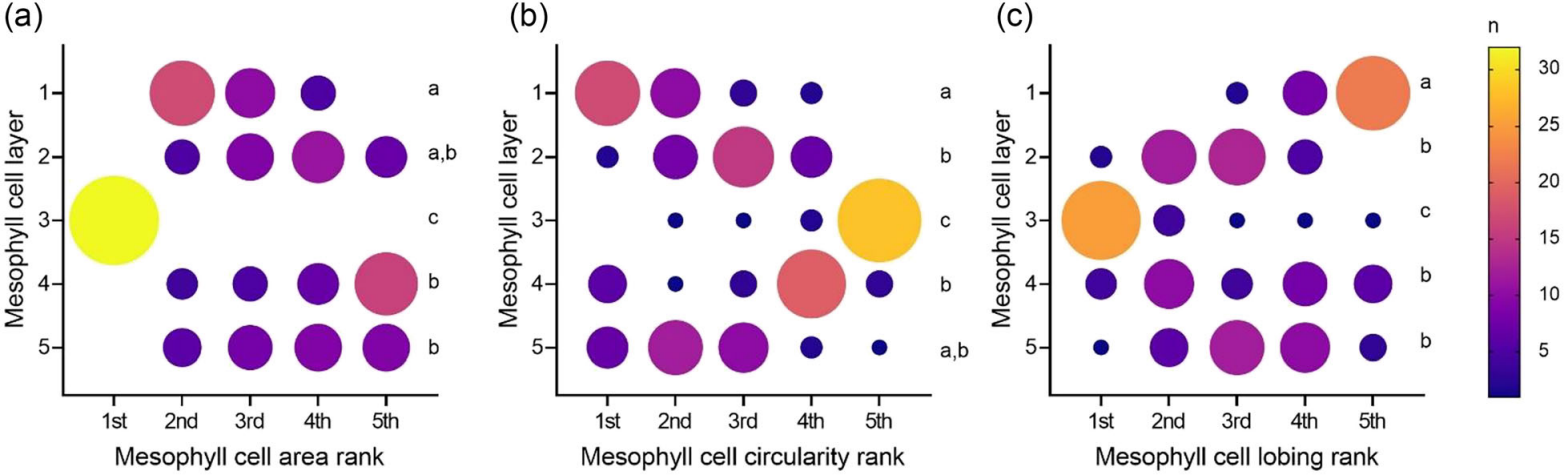
Rank frequencies of different mesophyll cell characteristics show clear patterns within the mesophyll. Cell characteristics are ranked from first (highest) to fifth (lowest) for each individual from the six species (*n* = 32). Size and color of circles represent the frequency of the rank occurring in that cell layer. (a) Mesophyll cell area ranks vary significantly by cell layer (Friedman test statistic value = 78.52, *p* < .001), Layer 1 is significantly different from Layers 3, 4, and 5, and Layer 3 is significantly different from all other cell layers. (b) Mesophyll cell circularity ranks vary significantly by cell layer (Friedman test statistic value = 70.82, *p* < .001), Layer 1 is significantly different from Layers 2, 3, and 4, and Layer 3 is significantly different from all other cell layers. (c) Mesophyll cell lobing ranks vary significantly by cell layer (Friedman test statistic value = 68.66, *p* < .001), Layer 1 is significantly different from all other cell layers, and Layer 3 is significantly different from all other cell layers. (a–c) Dunn's multiple comparison test, *p* < .05, layers that do not significantly differ from each other share a letter.

**FIGURE 5:**
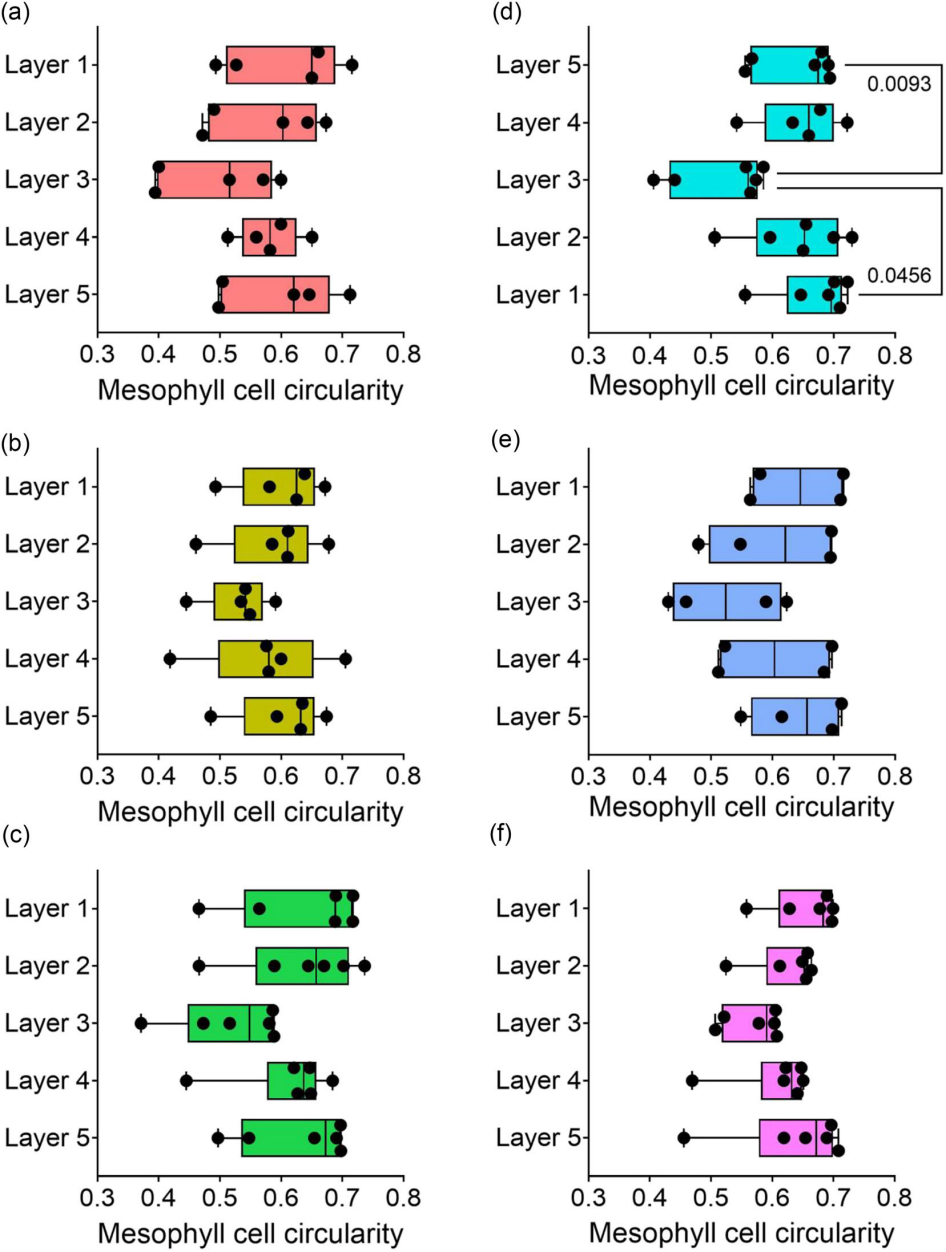
Layer 3 mesophyll cells have the lowest circularity in a range of rice varieties. (a) *O. sativa* MR220, (b) *O. sativa* MRQ76, (c) *O. sativa* Malinja, (d) *O. latifolia* , (e) *O. punctata* , and (f) *O. meridionalis* . One‐way ANOVA revealed a difference in cell area by layer in all lines: (a) *p* = .0081, *n* = 6; (b) *p* < .0001, *n* = 6; (c) *p* = .0368, *n* = 5; (d) *p* < .0001, *n* = 6; (e) *p* = .0009, *n* = 4; (f) *p* = .467, *n* = 6. Whiskers show min–max; average line represents the mean. Multiple pairwise comparisons (Tukey) are shown, with *p* values when lower than .05 (*n* = 4–6).

**FIGURE 6:**
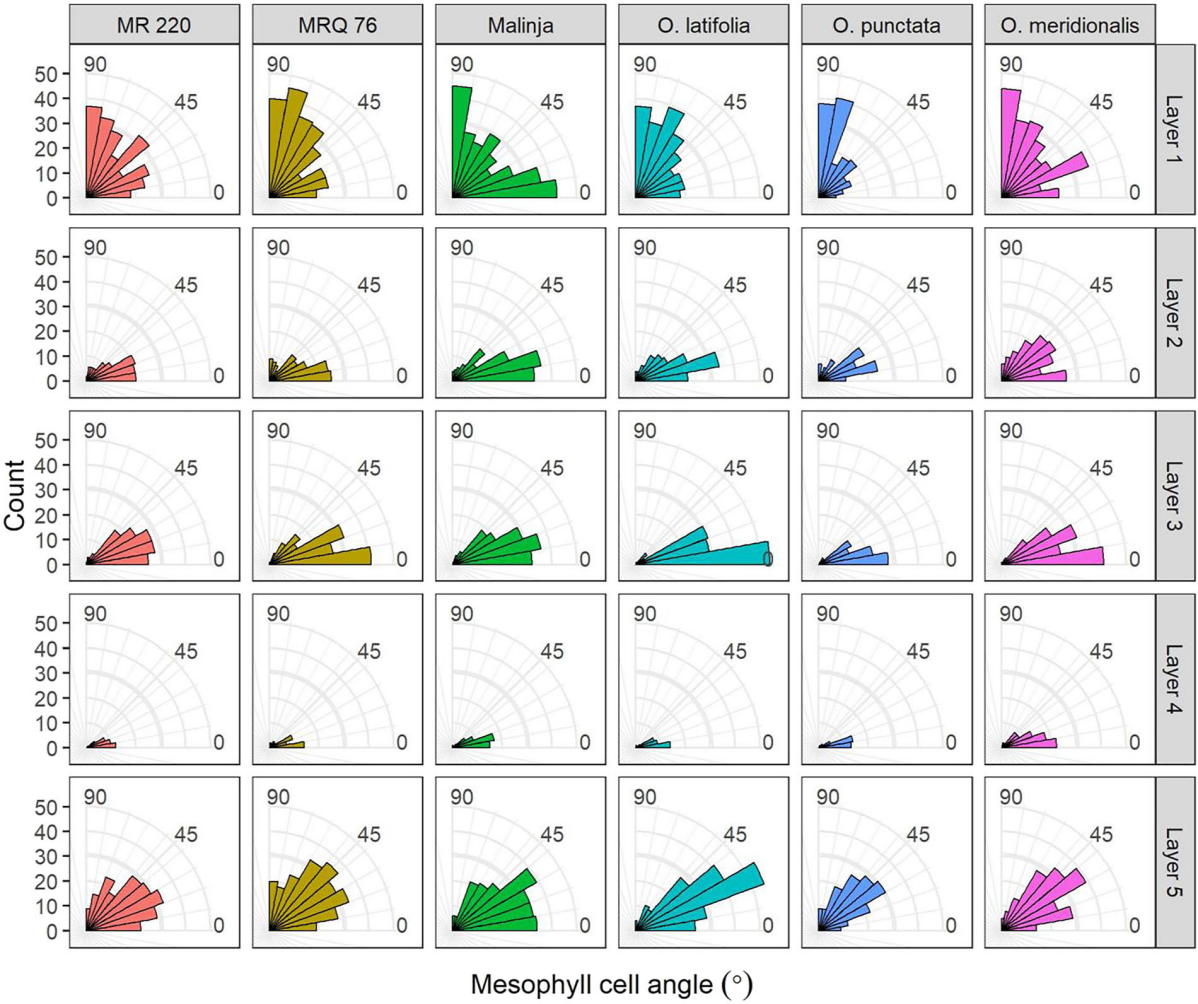
The long axis of Layer 3 mesophyll cells has a predominantly horizontal orientation in a range of rice varieties. The longest axis of cells in the internal mesophyll layers (2–4) is more horizontal than the layers adjacent to the epidermis. Cells in Layer 1 (adaxial) have a fairly random distribution of cell angle, and Layer 5 cells (abaxial) are most commonly at an angle of 30–50°.

Samples for Technovit® sectioning (Figure 1) and fresh transverse hand sections (Figures 2, 3, 4, 5, 6) were fixed in 1:4 acetic anhydride:ethanol for 48 h and then transferred to 70% ethanol. Hand sections were cleared in chloral hydrate saturated lactic acid for 2 h at 70°C and then stained for 20–30 s with 0.05% Toluidine Blue O. Technovit® samples were embedded in Technovit® 7100 resin and sectioned at 8 μm using a Leica Microtome and then stained for 20 s with Toluidine Blue O. All images were observed using an Olympus BX51 light microscope, with the 40× objective, Olympus DP71 camera, and Cell B imaging software. Regions of interest were between the first and second major veins out from the mid vein, between two minor veins.

Mesophyll cell image analysis was performed in FIJI (ImageJ 5.3g) software using an in‐house macro. The mesophyll layers were identified relative to their position in the leaf (Figure 1a). Layer 1 was identified as directly below the upper epidermis and bulliform cells, Layer 3 linking the middle of the left and right minor vein, Layer 5 directly above the lower epidermis, Layer 2 between Layers 1 and 3, and Layer 4 between Layers 3 and 5. Every cell within the layer was outlined by hand, and area (μm^2^), perimeter (μm), circularity, cell length (Feret), cell width (MinFeret), convex hull perimeter (μm), and cell angle (FeretAngle) measurements were taken. Mesophyll cell lobing was calculated as cell perimeter divided by convex hull perimeter, FeretAngle measurements were taken so that 0° corresponds to a horizontal line drawn between the minor veins in the image, and 90° is perpendicular to that line (see Figure S1). Cell projection images were created using an in‐house FIJI macro ‐ each cell was orientated so that the 0° line was horizontal, and then cell outlines were superimposed. For IR64 (Figure 1), leaf sections from eight plants were imaged. For the remaining rice species/varieties, leaf sections from four to six different plants were imaged. From each biological repeat, four images were analyzed.

### 2.3 Computational modeling

**FIGURE 7:**
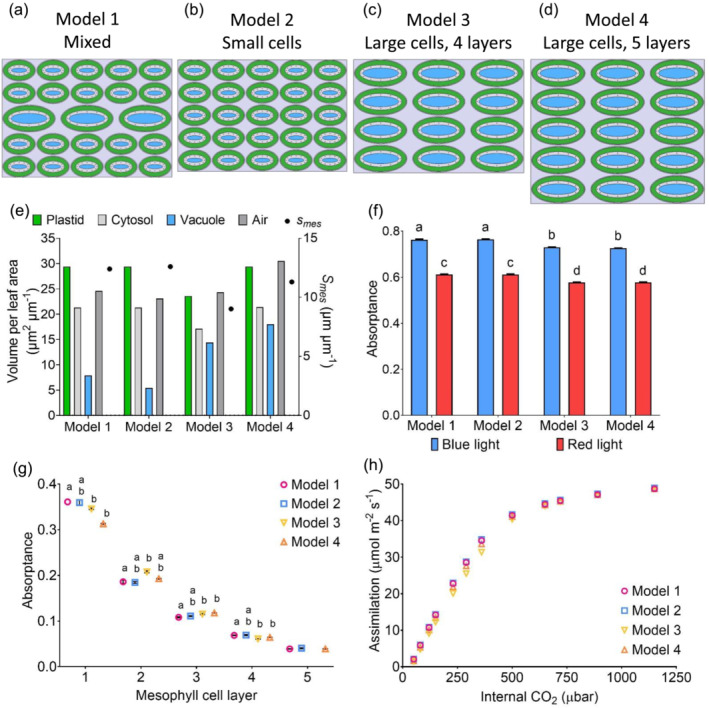
CO_2_ and light move differently through four simplified mesophyll tissue models. Four cell tissue layer models were designed, green represents plastid, pale gray is the cytosol, darker gray is air, and dots are mitochondria: (a) Model 1 has larger cells in the middle layer (Layer 3), (b) Model 2 has five layers of small cells, (c) Model 3 has four layers of large cells, and (d) Model 4 has five layers of large cells. Models 2 and 3 are the same leaf thickness. (e) *S*_*mes*_ and the proportions of different cell elements in the four models. Models 1, 2, and 4 have the same plastid and cytosol volume. (f) Total red and blue light absorptance is higher in Models 1 and 2 than Models 3 and 4 ‐ mean with SEM. Two‐way ANOVA, *p* < .0001, Tukey multiple comparison ‐ different letters represent significantly different values, *p* < .0001, *n* = 3. (g) Blue light absorptance in each cell layer of the four models ‐ mean values with SEM. Individual one‐way ANOVA performed for each cell layer ‐ Layers 1–4, *p* < .001; Layer 5, ns. Tukey multiple comparison ‐ different letters represent significantly different values, *p* < .05, *n* = 3. (h) Assimilation/internal CO_2_ (*C*_*i*_) curves are very similar for the four models. Mean values, *n* = 3. SEM is too small for error bars to show.

Four simplified models of mesophyll cell packing were designed (Figure 7a–d). Cell length and cell width (based on measurements from *O. latifolia*) were designed so that total length of three large cells in a layer equaled the total length of five small cells, and total leaf thickness of four large cells equaled the total leaf thickness of five small cells (Figure S2). Model 1 adopted a mix of two cell types with larger cells in its middle layer. Model 2 was generated by replacing the middle layer in Model 1 with small cells, and Model 3 had three layers of large cells, resulting in the same leaf thickness as Model 2. Model 4 had five layers of large cells. The thickness of the plastid layer and vacuole layer in both cell sizes was calculated to maintain plastid and cytosol volume between a layer of larger cells and a layer of small cells. Model 4, therefore, had the same plastid and cytosol volume as Model 1 and Model 2 (Figure 7e).

With the constructed leaf architecture, light propagation inside the leaf was simulated by a Monte‐Carlo ray tracing algorithm (Govaerts et al., 1996; Xiao et al., 2016, 2023). Due to the lack of epidermal cells in these simplified models, diffuse incident rays were emitted onto the upper boundary as the light source. Density of rays was tested to ensure the convergence of the simulations. Light absorptance of each chloroplast under blue and red light was simulated and applied to the later calculation of carboxylation rate for the process of CO_2_ reaction and diffusion. Details of the ray tracing algorithm and a list of related parameters can be found in Data S2.

CO_2_ reaction and diffusion inside the leaf were simulated by a partial differential system (Tholen & Zhu, 2011; Xiao et al., 2023; Xiao & Zhu, 2017). A constant CO_2_ concentration ([CO_2_]) was set to the upper and lower boundaries, representing [CO_2_] in the substomatal cavity, that is, C_i_. Inside the compartments of air space, cytosol, chloroplast, mitochondria, and vacuole, reaction–diffusion processes of CO_2_ were modeled by the following equations:

(E1) $$\left\{\begin{array}{c} D_{c}\cdot r_{f,i}\cdot \nabla^{2}C=f+h-r_{d}-r_{p}\\ D_{b}\cdot r_{f,i}\cdot \nabla^{2}B=-h \end{array}\right.$$

where *C* (mol m^−3^) and *B* (mol m^−3^) are the concentrations of CO_2_ and HCO_3_^−^, respectively. *D*_*c*_ (m^2^ s^−1^) and *D*_*b*_ (m^2^ s^−1^) are the liquid‐phase diffusion coefficients of CO_2_ and HCO_3_^−^ in water correspondingly. *r*_*f,i*_ is a dimensionless factor representing the change of the diffusion coefficient relative to free diffusion in water in different compartments. $\nabla^{2}C$ is the Laplace operator that equals $\frac{\partial^{2}C}{\partial x^{2}}+\frac{\partial^{2}C}{\partial y^{2}}$. While on the right‐hand side of the equation, *f* is volumetric carboxylation rate (mol m^−3^ s^−1^), *h* is hydration rate from CO_2_ to HCO_3_^−^ catalyzed by CA, *r*_*d*_ is volumetric respiration rate, and *r*_*p*_ is volumetric photo‐respiration rate. In addition, these terms were distributed differently in each compartment, for example, in the cytosol *f* = *r*_*d*_ = *r*_*p*_ = 0, in the chloroplast *r*_*d*_ = *r*_*p*_ = 0, and in mitochondria *f* = 0. The volumetric carboxylation rate and photo‐respiration rate were calculated based on the Farquhar‐von Caemmerer‐Berry model (Von Caemmerer, 2013). Details of the reaction–diffusion system and parameters used can be found in Data S2.

## 3 RESULTS

To investigate whether there was a pattern of mesophyll size and shape, mesophyll cells were assigned to different layers (1 to 5) within the leaf (Figure 1a). Layers 1 and 5 indicate the mesophyll cells immediately adjacent to the upper and lower epidermal cells, respectively (orange and dark blue cells). At the position of the bulliform cells, only three layers are present: Layers 1 and 5, and a middle layer which is defined here as Layer 3 (green). Outside the position of the bulliform cells, a layer between Layers 1 and 3 is generally present, defined as Layer 2 in light blue. At the extremities of the mesophyll adjacent to the vascular bundles, an incomplete layer of cells generally arises between Layer 3 and Layer 5, which is defined here as Layer 4 (yellow). Mesophyll cells were assigned to the different cell layers, and then the cells were analyzed for a range of quantitative parameters linked to size and shape (Figure 1b–f). With respect to size, cell area varied significantly between layers (Figure 1b, one‐way ANOVA, *p* < .0001, *n* = 8), with cells in the middle layer (Layer 3) being more than 30 μm^2^ larger on average than cells in every other layer (Tukey multiple comparison test, *p* < .002–*p* < .0001, *n* = 8). With respect to cell shape, analysis of circularity revealed that the cells in Layer 3 were significantly less circular than cells in the other layers (Figure 1c, one‐way ANOVA, *p* < .0001, *n* = 8, Tukey multiple comparison test, *p* < .005–*p* < .0001, *n* = 8). The most circular cells were in Layers 1 and 5 (sub‐epidermal cells) with an average circularity 0.1 higher than those in Layer 3, and cells in Layers 2 and 4 were intermediate (mean circularity levels 0.05 higher than Layer 3). Rice mesophyll cells are characterized by being highly lobed (Sage & Sage, 2009); therefore, this trait was also quantified, using a calculation of the ratio of actual cell perimeter to the minimal energy circumference of each cell as a measure of this parameter. These results revealed a variation in cell lobing between layers (Figure 1d, one‐way ANOVA, *p* < .0001, *n* = 8) with Layer 1 (upper sub‐epidermis) being distinguished by having cells that were less lobed than other layers in the mesophyll (Tukey multiple comparison test, *p* = .001–*p* < .0001, *n* = 8).

The differences in mesophyll cell size and shape between layers can be visualized by projecting cell outlines on top of each other (Figure 1e). In this visual analysis, the larger size of the Layer 3 cells was obvious. In addition, this analysis suggested that the Layer 3 cells were also distinctive in shape, with the long axis being more horizontal than cells in other layers. To investigate this, measurements were made of the angle of the long axis of individual mesophyll cells in each layer of the mesophyll (Figure 1f). These data generally substantiated the impression obtained from Figure 1e; that is, the Layer 3 cells had a long axis, which was predominantly aligned with the horizontal axis, which was also true for Layer 2 and Layer 4 cells. This equates to the mediolateral plane of the rice leaf, at right angles to the long axis of the leaf. The sub‐epidermal mesophyll cells in Layer 1 displayed a greater variation in cell axis orientation, and Layer 5 cells were mostly orientated between 20° and 50°.

In order to establish whether this pattern of mesophyll cell size and shape in IR64 leaves was reflected in the wider rice family, the mesophyll was studied in a range of rice varieties, including three cultivated *Oryza sativa* Indica variants (MRQ76, MR220, and Malinja) and three wild species (*O. latifolia*, *O. punctata*, and *O. meridionalis*). These variants showed a range of plant structure and size (Figure S3).

An overview of cell size and shape provided by mesophyll cell projections in the different layers and genotypes suggested that Layer 3 was distinct from the other cell layers, in terms of both relative size and cell orientation within a genotype (Figure 2). Quantitative analysis supported these visual impressions. For example, mesophyll cell size varied by layer in all the varieties analyzed (Figure 3, one‐way ANOVA *p* < .05–*p* < .0001, *n* = 4–6). In order to better understand the patterns within the data, a Friedman non‐parametric test was used, where the mean values for each cell layer were ranked from first (highest) to fifth (lowest) for each individual plant across the six varieties. The frequency of that rank occurring was plotted (Figure 4), and the likelihood of the values being distributed in a non‐random fashion was calculated. The rankings of mesophyll cell size by layer were highly significant (Friedman test statistic value = 78.52, *p* < .001, *n* = 32) showing that this pattern was strongly conserved. Notably, the mean cell size in Layer 3 was ranked first (meaning that these cells were largest) for every individual plant (Figure 4a). Layer 1 cells were most often ranked as the second largest layer of cells, ranking significantly lower than Layer 3 and higher than Layers 4 and 5 (Dunn's multiple comparison test, *p* < .05, *n* = 32).

As with IR64, Layer 3 cells had the lowest mean circularity value compared to other cell layers in all variants analyzed (Figure 5), although within a variant only the *O. latifolia* leaves displayed a significantly lower value of cell circularity compared to cells in other layers within the leaf (Figure 5d, Tukey, *p* < .05, *n* = 6). Considering the ranking of cell circularity across the 32 individual plants, Layer 3 cells were most often ranked fifth, or least circular, of the five mesophyll cell layers (Figure 4b, Friedman test statistic value = 70.82, *p* < .001, Dunn's multiple comparisons test *p* < .01, *n* = 32). Layer 1 sub‐epidermal cells also had a significantly different ranking pattern to Layers 2, 3, and 4, with a predominance for ranking as the most circular (Dunn's multiple comparisons test *p* < .01, *n* = 32).

For cell lobing, although there was a trend for Layer 3 cells to have higher lobing values, the differences observed were not statistically significant at *p* = .05 (Figure S4). However, while investigating the ranking of cell lobiness between layers, a pattern was evident (Figure 4c, Friedman test statistic value = 68.66, *p* < .001), with Layer 3 cell rankings significantly skewed towards first (most lobed) compared with all other cell layers (Dunn's multiple comparisons test *p* < .01, *n* = 32). Layer 1 cells also showed a unique pattern of rankings, significantly different to all other cell layers, ranking fifth (or least lobed) for many individual mesophylls (Dunn's multiple comparisons test *p* < .01, *n* = 32).

When the orientation of individual mesophyll cells was measured according to layer, Layer 3 was distinguishable in all variants as having cells whose long axis lay predominantly in the medio‐lateral plane of the leaf (Figure 6). Layers 2 and 4 showed similar orientation, although slightly less striking. This was distinct from the pattern seen in the sub‐epidermal layers ‐ Layer 1 mesophyll cell orientation was more evenly distributed, reflecting their increased circularity, with a slight tendency towards more vertical cell orientation in some varieties (MRQ76, *O. latifolia* and *O. punctata*). It is important to note that these cells follow the perimeter of bulliform cells which may also account for the wide range of axiality angle. The cells on the abaxial side of the mesophyll (Layer 5) showed a predominance towards a 40° angle, which could reflect their broadly rectangular shape (evident in Figure 2).

To investigate how the pattern of mesophyll cell size revealed by this analysis might influence leaf photosynthetic performance in terms of basic light absorption and carbon assimilation rate, an initial 2D modeling approach was utilized. Four simplified models of mesophyll cell packing were generated, as shown in Figure 7a–e. Model 1 was most representative of mesophyll described in this study, with five layers, in which the middle layer (Layer 3) was characterized by having relatively large cells (Figure 7a). Model 2 had the same number of layers as Model 1, but the cells were all uniform and relatively small (Figure 7b). Consequently, the Model 2 leaf was slightly thinner than Model 1. Models 3 and 4 were made up entirely of relatively large cells with four and five layers, respectively (Figure 7c,d). Model 4 was therefore slightly thicker than Models 1, 2, and 3. Each cell was modeled to have a proportion of plastid inversely proportional to cell size and a proportion of cytosol proportional to cell size, reflecting the findings of Sage and Sage (2009). Cell wall thickness and mitochondrial size were the same in all models. As a consequence of these parameters and packing, Models 1, 2, and 4 had the same plastid and cytosol volumes (Figure 7e), but differed in their leaf thickness. Model 3 had the lowest plastid and cytosol volume. The amount of cell wall in contact with the air (*S*_*mes*_) was very similar in Models 1 and 2, lowest in Model 3, and intermediate in Model 4 (Figure 7e).

When the model leaves were supplied with direct incident light from the adaxial surface, Models 1 and 2 had higher total light absorptance than Models 3 and 4, for both red and blue wavelengths (Figure 7f). However, Models 3 and 4 (consisting of entirely larger cells) allowed more light to travel further into the leaf, with significantly higher absorptance than Model 1 in cell layers 3 and 4 (Figure 7g). Conversely, Model 1 had the highest light absorption in Layer 1. When photosynthetic performance was modeled, there was little to distinguish the models, although Models 3 and 4 performed slightly less well during the Rubisco‐limited initial slope of the simulated A/Ci curve (Figure 7h). Model 3, with the lowest volume of plastid and lowest light absorptance, had the lowest assimilation rate at low internal CO_2_ concentration.

## 4 DISCUSSION

### 4.1 Rice mesophyll displays a conserved pattern of size and shape

The analysis reported here indicated that rice leaves from a range of genotypes and variants display a conserved pattern of cellular architecture. In particular, mesophyll cells in the middle (Layer 3) of the leaves were larger than mesophyll cells in other layers (Figures 1b, 3, and 4a), had the lowest degree of circularity (Figures 1c, 4b, and 5), and tended to have a cell long axis oriented orthogonal to the longitudinal axis of the leaf, thus aligning with the medio‐lateral plane of the leaf (Figures 1e and 6). A distinctive pattern of cell axiality was also observed in the most adaxial mesophyll layer (Layer 1) where cells displayed a much wider range of long axis orientation than cells in the other layers of the mesophyll. These cells were also most likely to be ranked as the least lobed (Figures 1d and 4c). These observations contrast with a widely accepted text‐book view that in monocots, mesophyll cell size and shape are distributed uniformly within the leaf (Beck, 2010; Pyke, 2012).

There have been previous suggestions that this view of grass leaf anatomy might be an over‐simplification of the true situation. For example, in the original paper highlighting the potential importance of cell lobing in rice (Sage & Sage, 2009), the authors showed that the cells towards the middle of the mesophyll tended to be more elongated and had a larger vacuole and a lower proportion of chloroplast by volume than cells nearer the outside of the leaf. Our data build and extend this view to show that there is a clear and consistent pattern in a range of rice species in which cells in the middle layer are significantly larger than cells in other layers of the mesophyll, have a distinct shape (higher circularity), and display a restraint in cell axiality absent in cells in other layers of the leaf. The idea that grass leaves such as rice have a generally uniform pattern of mesophyll cellular architecture contrasts with eudicots where the distinction of the palisade and spongy mesophyll has long been established (Beck, 2010; Chonan, 1978; Esau, 1965; Pyke, 2012). It is interesting to note that even in the eudicot system, recent work using microCT imaging has revealed that the spongy mesophyll is more organized than was previously thought (Borsuk et al., 2022). The data presented in this article thus fit to a trend that leaf cellular architecture may be more structured than is widely accepted. This raises the question of how the rice mesophyll pattern arises and what, if any, advantage this arrangement of cells conveys to the leaf.

With respect to development, Zeng et al. (2016) showed that the middle layer of the rice mesophyll (Layer 3 in this paper) is derived from the L3 cells of the shoot apex, whereas the cells neighboring the epidermal cells are derived from L2 cells. The Layer 3 cells are thus likely to be clonally distinct, so their size, shape, and axiality might, theoretically, reflect ontogeny. A more precise analysis of cell size and shape across the emerging layers in the developing rice leaf would help test this possibility. An alternative (though not exclusive) hypothesis is that the cellular pattern across the adaxial/abaxial axis of the leaf is linked to specific function, for example, photosynthesis. In many eudicot leaves, the mesophyll cells that form the palisade layer are vertically aligned and cylindrical in shape to aid light penetration to the lower spongy mesophyll (Holloway‐Phillips, 2019; Terashima et al., 2016). It is possible that the more vertical orientation of the cells in Layer 1 and, to a lesser extent, Layer 5 of the rice mesophyll (the external layers of the mesophyll) has a similar role in directing light towards the more internal mesophyll of the leaf. The challenge here is that variation in cell size and shape across the mesophyll probably reflects a complicated trade‐off between optimizing surface/area to volume for gas exchange, the optimum spread of material for light absorption, and the investment costs (carbon, nitrogen, energy) in building a leaf, as has been explored by (Earles et al., 2019).

To make an initial analysis of this problem, a modeling approach was taken. The results (Figure 7) suggest that the cellular pattern observed in rice, with larger cells in the middle layer of the leaf, led to a slight increase in light absorption in the outer, adaxial layer of the mesophyll. This can be explained by the stronger sieve effect (as in Terashima et al., 2009) in the larger cells due to the chloroplasts being spread more sparsely. Nevertheless, the overall impact on carbon assimilation rate is likely to be minimal. Of course, the model used here is a gross simplification of reality, so this conclusion should be treated with some caution, but it does suggest that a role for the observed mesophyll cell pattern in photosynthesis may not be trivial (if it exists). This point raises the question of whether the specific cell pattern in the middle cell layer of the leaf might have another role. For example, it might reflect a mainly mechanical role in supporting the leaf lamina. Alternatively, it is interesting to note that a by‐product of the pattern is that there were fewer cell boundaries in the lateral plane of the leaf connecting adjacent veins. If Layer 3 has a role in transporting molecules to and from vascular bundles, a trait of fewer cell boundaries might be advantageous.

Finally, our findings have implications (both negative and positive) for related research in the broader area of rice research. First, many studies taking a comparative approach to leaf structure in grasses use the middle layer of the mesophyll as an easily identifiable region to sample, thus decreasing the work‐load involved in often largescale analyses (e.g., Chatterjee et al., 2016; Ouk et al., 2020). Our data suggest that, unfortunately, the cells in this layer are in some ways atypical of the mesophyll as a whole. On the other hand, there is significant interest in engineering rice leaves to instill a major shift in photosynthesis (C_4_ photosynthesis) ‐ with decreasing the number of mesophyll cells between vascular bundles as a key aim (Ermakova et al., 2020). Our data indicate that, due to their size and axiality, the middle layer of the rice mesophyll already provides the fewest cells between neighboring veins. Driving this anisotropic growth further is an avenue to explore, which might contribute to achieving this leaf engineering goal.

## AUTHOR CONTRIBUTIONS

Jen Sloan, Saranrat Wang, Qi Yang Ngai, Jodie Armand, and Matthew J. Wilson performed the experiments; Yi Xiao performed the computational modeling; Jen Sloan, Saranrat Wang, Qi Yang Ngai, Yi Xiao, Jodie Armand, Matthew J. Wilson, Xin‐Guang Zhu, and Andrew J. Fleming interpreted the results and wrote the paper, with contributions from all authors. Andrew J. Fleming designed the study and led the project.

## CONFLICT OF INTEREST STATEMENT

No conflict of interest declared.

### PEER REVIEW

The peer review history for this article is available in the Supporting Information for this article.

## Supporting information


**Data S1.** Peer review.

**Data S2.** Supplementary methods.
**Table S1.** Refractive indexes and specific absorption coefficients used in the ray tracing, modified from the supplementary worksheet in Xiao et al. (2016).
**Table S2.** Biophysical and biochemical parameters used in the reaction–diffusion equations of CO_2_ and HCO_3_^−^.

**Figure S1:** Measurement of mesophyll cell lobing and orientation.
**Figure S2:** Measurements of large and small cells used in leaf tissue models.
**Figure S3:** Six different varieties of rice used in Figures 2, 3, 4, 5, 6 and Figure S4 show a range of plant structure and size.
**Figure S4:** Layer 1 mesophyll cells always have the lowest lobing value across a range of varieties.

## Data Availability

The data that support the findings of this study are available from the corresponding author upon reasonable request.
