# Supplementary material for: Conserved cellular patterning in the mesophyll of rice leaves
Source: Plant Direct. 2023 Dec 4;7(12):e549. doi: 10.1002/pld3.549 (PMC10695703; doi:10.1002/pld3.549)
Supplement: Supplementary file 2 — Data S2. Supplementary methods. Table S1. Refractive indexes and specific absorption coefficients used in the ray tracing, modified from the supplementary worksheet in Xiao et al. (2016). Table S2. Biophysical and biochemical parameters used in the reaction–diffusion equations of CO2 and HCO3 −. [file PLD3-7-e549-s002.docx]

**Supplementary Methods**

**A. The Monte-Carlo ray tracing algorithm to simulate light propagation**

With a ray hit on an interface between two different substance, reflection and refraction is calculated following the Fresnel equation (see refractive indexes in **Table S1**). Strictly speaking, ray tracing should continue with the reflected ray and refracted ray. However, in order to avoid the computational complexity brought by this exponential increase of number of rays to trace, a Monte-Carlo algorithm is integrated so that only either the reflected or refracted ray is randomly traced further with the full energy of the incident ray. The possibility of this selection is proportional to the intensity of reflected and refracted rays. Therefore, if this Monte-Carlo procedure is simulated repetitively, then the mean result of massive simulations will approximate the strict ray tracing algorithm without the Monte-Carlo procedure. Meanwhile, when a ray travels inside a substance, light absorptance is calculated by the Beer-Lamber law (see absorption coefficients in **Table S1**). For a ray hit the left or right boundaries of the model, it is reflected with full energy and the ray tracing can be continued until it hits the upper or lower boundary, which will be summarized as the leaf reflectance and transmittance.

| Compartment | refractive index | s.a.c under 475nm | s.a.c under 625nm |
| --- | --- | --- | --- |
| air | 1.000 | / | / |
| cell wall | 1.415 | 4.38 (cm2 g-1) | 4.38 (cm2 g-1) |
| cytosol | 1.353 | 1.14e-4 (cm-1) | 2.834e-3 (cm-1) |
| chloroplast | 1.511 | 4.26e4 (cm2 g-1) | 2.34e4 (cm2 g-1) |
| vacuole | 1.333 | 1.14e-4 (cm-1) | 2.834e-3 (cm-1) |

**Table S1. Refractive indexes and specific absorption coefficients used in the ray tracing, modified from the supplementary worksheet in Xiao et al. (2016).**

To simulate the An-Ci curves, the light source was set to be 10% blue light and 90% red light which is frequently used during the measurement with LI-6800 (LI-COR, Lincoln, USA).

**B. The partial differential system to simulate CO2 reaction-diffusion**

With a constant [CO2] set to the upper and lower boundary, CO2 molecules diffuse in the gas phase in the intercellular air space. Then on the outer side of cell wall, [CO2] in liquid phase was converted from CO­2 partial pressure based on Henry’s law.

Internal surfaces such as cell wall and membrane, chloroplast envelope, and membrane of mitochondria and vacuole were modelled as a thin diffusion barrier with a given permeability for CO2,

$$n\cdot(-D\nabla C)=P_{CO2}(C_{1}-C_{2})$$

where *n* is the normal vector of the surface. The left-hand side of the equation is the diffusive flux of CO2 across the surface. *PCO2* (m s-1) is the permeability of the boundary to CO2, and *C1* and *C2* (mol m-3) are the concentration on both sides of the surface. For HCO3-, these inner boundaries were modelled in the same way except that the surfaces representing cell wall and cell membrane were set to be impermeable to HCO3-.

The volumetric carboxylation rate *f* in equation E1 in the main text is calculated based on the Farquhar-von Caemmerer-Berry model:

$$\begin{matrix} f=\min(f_{c},f_{j}) \\ \text{ }=\min(\frac{k_{c}X_{c}C}{C+K_{m}},\frac{jC}{4C+8\Gamma^{*}}) \end{matrix}$$

Where *fc* (mol m-3 s-1) is volumetric Rubisco limited carboxylation rate, and *fj* is (mol m-3 s-1) volumetric RuBP-regeneration limited carboxylation rate. *kc* (s-1) is Rubisco turnover rate, and *Xc* (mol m-3) is the Rubisco concentration. *C* (mol m-3) is the CO2 concentration, *Km* (mol m-3) is the effective Michaelis-Menten constant under specific oxygen concentrations, *j* (mol m-3) is the volumetric electron transport rate and *Г** (mol m-3) is the compensation point of chloroplastic [CO2] in the absence of respiration.

The electron transport rate of *i*th chloroplast $j^{(i)}$ is calculated from a hyperbolic equation,

$$j^{(i)}=\mathrm{minh}(j_{i}^{(i)},j_{m}^{(i)})=\left\{j_{i}^{(i)}+j_{m}^{(i)}-\left[\left(j_{i}^{(i)}+j_{m}^{(i)}\right)^{2}-4\theta j_{i}^{(i)}j_{m}^{(i)}\right]^{0.5}\right\}/2\theta$$

where $j_{i}^{(i)}$, i.e. the light limited rate of PSII electron transfer rate of *i*th chloroplast, is calculated by,

$$j_{i}^{(i)}=I\cdot ab^{(i)}\cdot\beta\cdot Y(II)_{LL}$$

where *I* is the incident irradiance, *ab*(*i*) is the light absorptance predicted from ray tracing, *β* is the proportion of absorbed light partitioned to PSII, and *Y(II)LL* is the conversion efficient of photosystem II from absorbed photons into e-.

The volumetric photorespiration rate *rp* in the mitochondria was calculated as integration over the chloroplast volume and assigned uniformly to mitochondria by dividing by the mitochondria volume (*Vm*) in that mesophyll cell (equation S5).

$$r_{p}=\frac{\iiint_{chl} \frac{f\Gamma^{*}}{C}dxdydz}{V_{m}}$$

The volumetric respiration rate *rd*, was calculated from the respiration rate per leaf area (*Rd*) by dividing by the mitochondria volume (equation S6).

$$r_{d}=\frac{R_{d}S}{\sum V_{m}}$$

The hydration rate *h* (mol m-3 s-1) was approximated by equation S7.

where *ka* (s-1) is the turnover rate of CA, *Xa* (mol m-3) is the concentration of CA, *H* (mol m-3) is the proton concentration, *Ka* (mol m-3) and *KHCO3* (mol m-3) are the Michaelis-Menten constants of hydration and dehydration, and *Keq* (mol m-3) is the equilibrium constant.

Parameters describing the diffusion of CO2 and HCO3-, and the reaction rate of hydration were the same as the default parameters used in Tholen and Zhu (2011), Xiao and Zhu (2017) and Xiao et al. (2023). Biochemical parameters related to photosynthetic metabolism were rounded from values in Xiao et al. (2023) fitted from measurements of the IR64 rice leaf. For the convenience of adopting these values to our 2D leaf model, leaf geometries in **Fig. 7A-D** were extruded to have certain depth. But, since we also constrained the boundary conditions for the front and back surfaces to be no-flux, the solution shows no diffusive fluxes of CO2 or HCO3- along the depth. This reaction-diffusion system was implemented and solved with COMSOL Multiphysics 5.3 (COMSOL, Inc. Stockholm, Sweden)

| Name | Symbol | Value | Units |
| --- | --- | --- | --- |
| CO2 diffusion coefficient in water | *Dc* | 1.83×10-9 | m2 s-1 |
| Cytosol viscosity relative to water | *ηc* | 2 | Relative to water |
| Stroma viscosity relative to water | *ηs* | 10 | Relative to water |
| Mitochondria viscosity relative to water | *ηm* | 10 | Relative to water |
| CO2 concentration | *C* | Calculated | mol m-3 |
| HCO3- diffusion coefficient in water | *Db* | 0.52×*Dc* | m2 s-1 |
| HCO3- concentration | *B* | Calculated | mol m-3 |
| Carboxylation rate | *f* | Calculated | mol m-3 s-1 |
| Carboxylase *Km* | *Kc* | 239 | μbar |
| Oxygenase *Km* | *Ko* | 266 | mbar |
| Volumetric electron transport rate | *j* | Calculated | mol m-3 s-1 |
| CO2 compensation point | *Г** | 1.35×10-3 | mol m-3 |
| Rubisco relative specificity | *Sc/o* | 3375 | - |
| Hydration rate | *h* | Calculated | mol m-3 s-1 |
| CA turnover rate | *ka* | 3×105 | s-1 |
| CA concentration cytosol | *Xa,c* | 0.5×*Xa,s* | mol m-3 |
| CA concentration stroma | *Xa,s* | 0.27 | mol m-3 |
| Proton concentration | *H* | 10-pH | mol m-3 |
| Cytosol pH | *pHc* | 7.3 | - |
| Chloroplast pH | *pHs* | 8 | - |
| Mitochondria pH | *pHm* | 8 | - |
| CA equilibrium constant | *Keq* | 5.6×10-7 | mol m-3 |
| CA hydration *Km* | *Ka* | 1.5 | mol m-3 |
| CA dehydration *Km* | *KHCO3* | 34 | mol m-3 |
| Volumetric respiration rate | *rd* | Calculated | mol m-3 s-1 |
| Volumetric photorespiration rate | *rp* | Calculated | mol m-3 s-1 |
| Maximal Rubisco carboxylation rate | *Vcmax* | 114 | μmol m-2 s-1 |
| Maximal electron transport rate | *Jmax* | 225 | μmol m-2 s-1 |
| Respiration rate under light | *Rd* | 1 | μmol m-2 s-1 |
| Convexity index | *θ* | 0.98 | - |
| Proportion of absorbed light partitioned to PSII | *β* |  | - |
| Conversion efficient of PSII from absorbed photons into e- | *Y(II)LL* |  | - |
|  | *β·Y(II)LL* | 0.6 | - |

**Table S2. Biophysical and biochemical parameters used in the reaction-diffusion equations of CO2 and HCO3-**
