# Supplementary material for: Conserved cellular patterning in the mesophyll of rice leaves
Source: Plant Direct. 2023 Dec 4;7(12):e549. doi: 10.1002/pld3.549 (PMC10695703; doi:10.1002/pld3.549)
Supplement: Supplementary file 3 — Figure S1: Measurement of mesophyll cell lobing and orientation. Figure S2: Measurements of large and small cells used in leaf tissue models. Figure S3: Six different varieties of rice used in Figures 2, 3, 4, 5, 6 and Figure S4 show a range of plant structure and size. Figure S4: Layer 1 mesophyll cells always have the lowest lobing value across a range of varieties. [file PLD3-7-e549-s001.pdf]

A

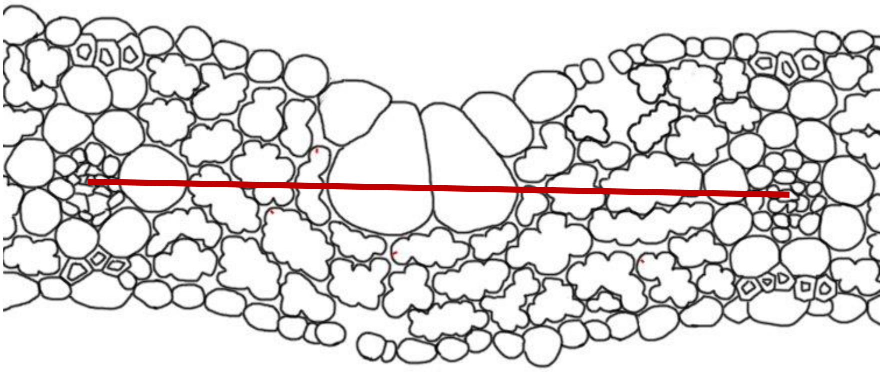

B

Cell perimeter	Convex hull perimeter	Lobing (cell perimeter/ convex hull perimeter)	Longest axis (Feret's diameter)	Feret Angle	Adjusted if FeretAngle > 90	Final angle
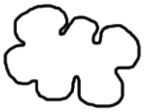	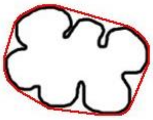	1.33	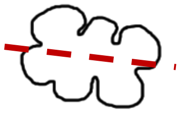	172°	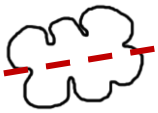	8°
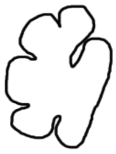	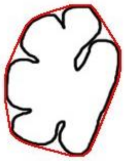	1.25	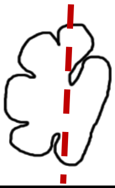	84°	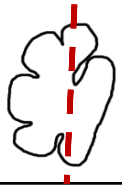	84°
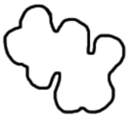	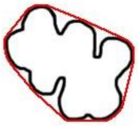	1.33	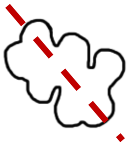	128°	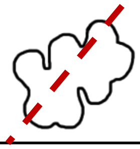	52°
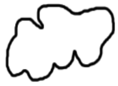	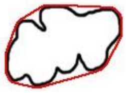	1.28	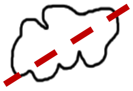	24°	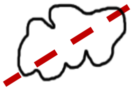	24°

Figure S1: Measurement of mesophyll cell lobing and orientation

A) A line was drawn between the two minor veins in each image. The angle of this line was measured and considered horizontal or 0°.

B) Cell perimeter and convex hull perimeter were measured in ImageJ. Lobing is calculated as cell perimeter/convex hull perimeter.

The FeretAngle measurement (0-180 degrees) is the angle between the Feret's diameter and a line parallel to the x-axis of the image. The horizontal angle was subtracted from this angle so that a cell angle of 0° is parallel to the line between the minor veins. If the FeretAngle is >180°, the angle was adjusted (180-FeretAngle) so that all angles were between 0 and 90° for ease of comparison. A cell with an angle of 90°is aligned with its longest axis vertical (or perpendicular to the line between the minor veins).

A

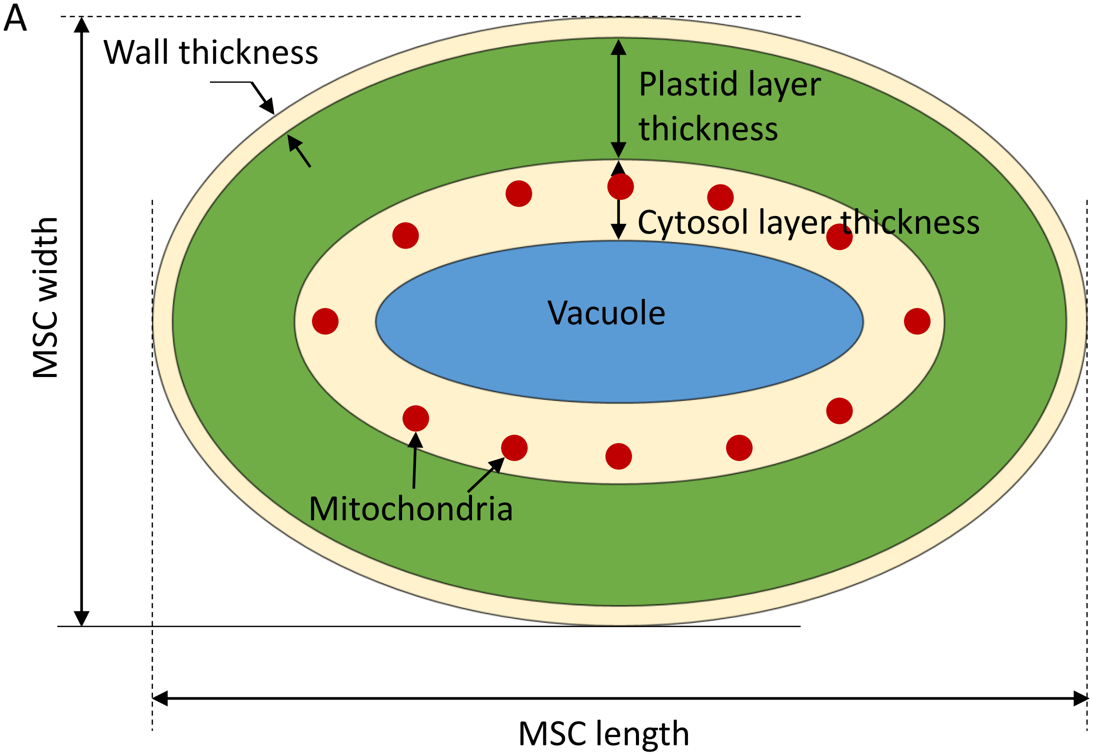

B

**Layer No.**	**Parameter**	**Value**
Small cells	MSC width	15 um
	MSC length	23 um
	Wall thickness	0.5 um
	Plastid layer thickness	3 um
	Cytosol layer thickness	2.5 um
	Distance between mitochondria	1
	Mitochondria radius	0.2
Large cells	MSC width	19 um
	MSC length	37 um
	Wall thickness	0.5 um
	Plastid layer thickness	3.14 um
	Cytosol layer thickness	2.23 um
	Distance between mitochondria	1
	Mitochondria radius	0.2

Figure S2: Measurements of large and small cells used in leaf tissue models
A) Detailed representation of each cell in the leaf tissue model. **B)** Different parameter measurements used for small and large cells in leaf tissue models

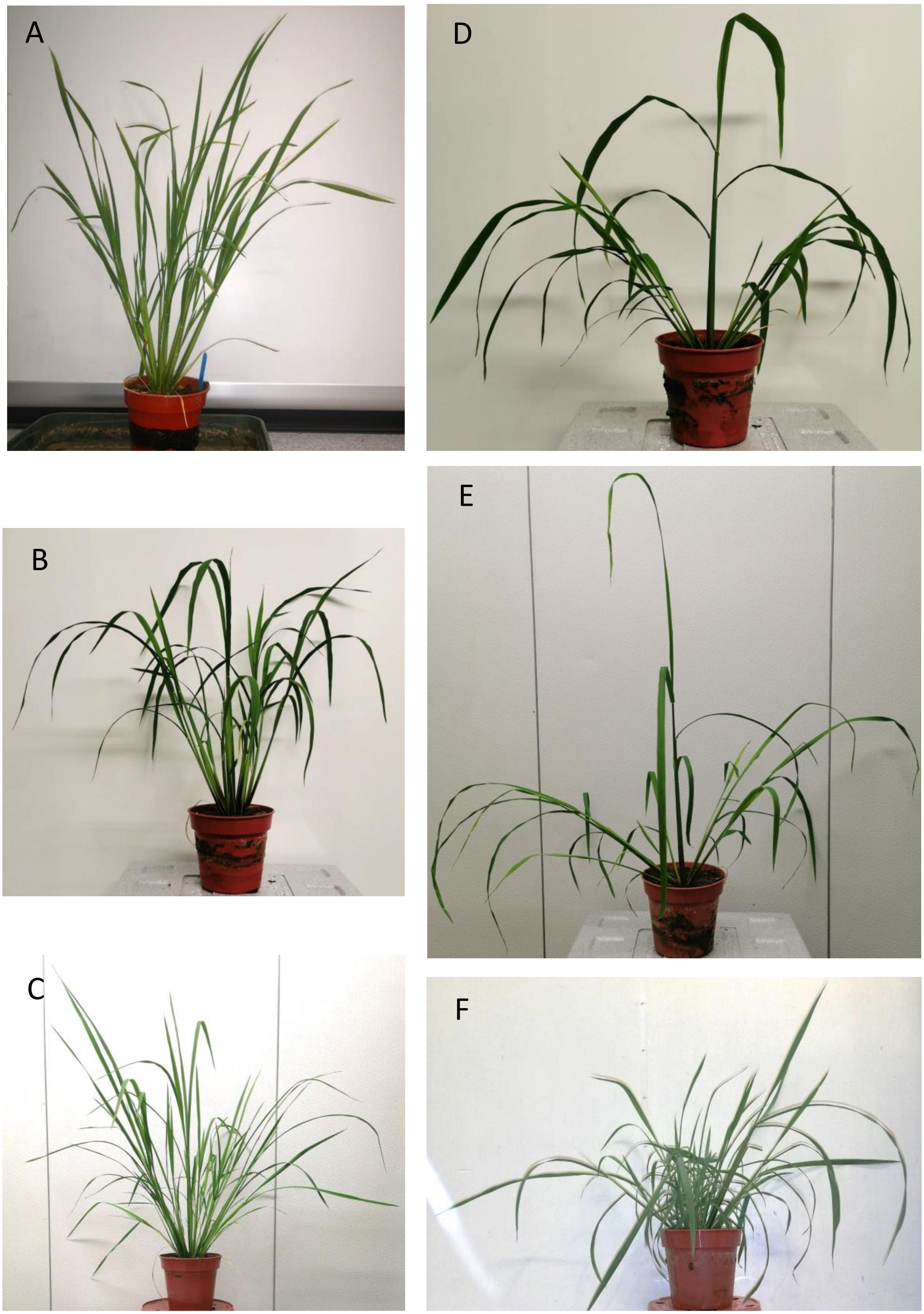

Figure S3: Six different varieties of rice used in Figures 2-6 and Supplementary Figure 4 show a range of plant structure and size
Plants pictured at 35 days old.
A) *Oryza sativa* (MR220), B) *Oryza sativa* (MRQ76), C) *Oryza sativa* (Malinja), D) *Oryza latifolia*, E) *Oryza punctata*, F) *Oryza meridionalis*

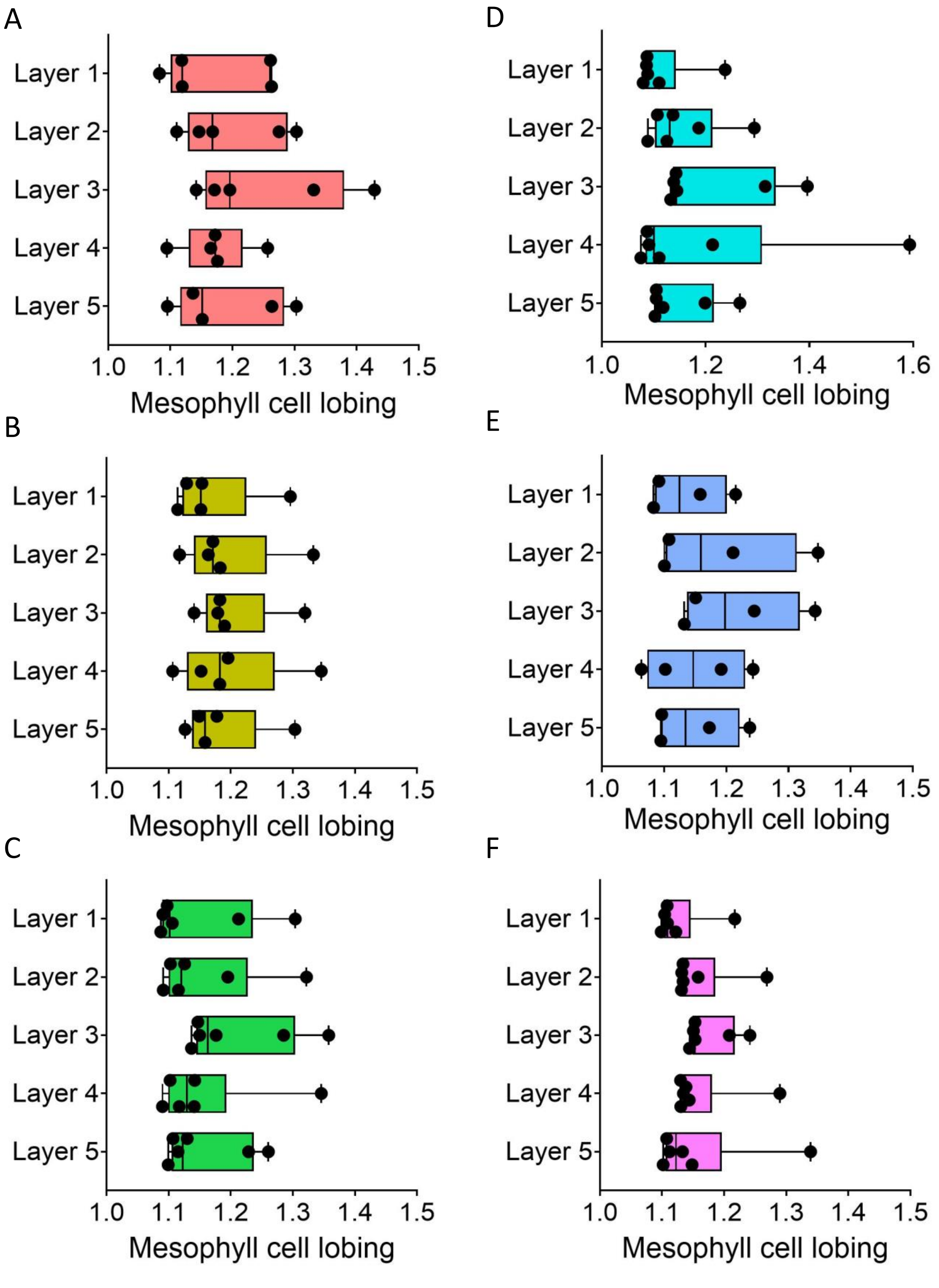

Figure S4: Layer 1 mesophyll cells always have the lowest lobing value across a range of varieties Mesophyll cell lobing from the middle of leaf 6 of 6 rice varieties – **A)** *O.s ativa* MR220, **B)** *O. sativa* MRQ76, **C)** *O. sativa* Malinja, **D)** *O. latifolia*, **E)** *O. punctata*, **F)** *O. meridionalis*. Note the different x axis scale in panel D. Whiskers show min-max, average line represents the mean. Cell lobing does not significantly vary across the abaxial/adaxial gradient. One way ANOVA, $p > 0.05$, $n = 4\text{-}6$.
