# Supplementary material for: Conserved cellular patterning in the mesophyll of rice leaves
Source: Plant Direct. 2023 Dec 4;7(12):e549. doi: 10.1002/pld3.549 (PMC10695703; doi:10.1002/pld3.549)
Supplement: Supplementary file 1 — Data S1. Peer review. [file PLD3-7-e549-s003.pdf]

Plant Direct
Open Access

Conserved cellular patterning in the mesophyll of rice leaves

Sloan Jen, Wang Saranrat, Ngai Qi Yang, Xiao Yi, Armand Jodie, Wilson Matthew J., Zhu Xin-Guang., Fleming Andrew J.

DECISION LETTER- ROUND 1

October 9, 2023

Dr. Jen Sloan
University of Sheffield
Sheffield, N/A
United Kingdom

RE: Conserved cellular patterning in the mesophyll of rice leaves

Dear Dr. Sloan:

Thank you for submitting to Plant Direct. All required reviews have been returned and we have now finished our evaluation of your manuscript. In light of the reviewers' and editor's comments, further revisions are needed before the paper can be accepted for publication in Plant Direct.

Please view the editors' and reviewers' comments below and use their suggestions as a guide while you work on your revision.

When uploading the revised version of this article, please be sure to include the following:

-A word document that contains your response to the reviewers. You should respond to each reviewer comment and note the changes made to the manuscript. If you do not agree with a reviewer's comment and choose not to make a suggested revision, please explain why. Please try to provide as complete an answer as possible to each reviewer's criticisms.
-A tracked changes document with each change highlighted
- A clean version of the latest version of the manuscript

Additional comments:

Please add the figure number for each figure in your figure file.

Please change the reference to Supp. Fig. 3 to Supp. Fig. S3 (see line 207).

Please change the reference to Supp. Fig. 4 to Supp. Fig. S4 (see line 233).

To upload your revision, please click the link below.
https://plantdirect.msubmit.net/cgi-bin/main.plex?el=A6Lr1cs7A5oPU1I4A9ftdO78ikVKE94sHVpSVRzInwZ

In order to provide as timely a service as possible, we ask that your revision is resubmitted within three months after receipt of this request. If an extension is needed, please send a request, along with a brief explanation, to the editorial office at plantdirect@wiley.com .

Please note that, in addition to publishing reviewer comments, the author's responses to review comments will also be published alongside the final version of the paper. If you would not like the author's responses to be published, please contact the editorial office at plantdirect@wiley.com .

Thank you very much for giving us an opportunity to review your work. I look forward to receiving the next version.

Sincerely,

Reggie Millwood

Gustavo MacIntosh

Editor, Plant Direct

--- Editor comments:
Dear authors,

Please add the figure number for each figure in your figure file.

Please change the reference to Supp. Fig. 3 to Supp. Fig. S3 (see line 207).

Please change the reference to Supp. Fig. 4 to Supp. Fig. S4 (see line 233).

--- Reviewer comments:
Reviewer #1:

Comments to the Author:

Title: Conserved cellular patterning in the mesophyll of rice leaves

Overview and general recommendation:
The manuscript deals with an important topic related to the conserved cellular patterning in the

mesophyll of rice leaves. The manuscript technically sounds well and shows high novelty. It is well written and shows the need for minor linguistic adjustments. In this regard, the needed adjustments are highlighted in "Minor comments" section. The use of past verbal tense is needed when reporting the findings of the study. The first voice form of the sentence shall be avoided and the impersonal form shall be adopted instead. The references used in both Introduction and Discussion sections shall be up-to-date (2010 and onwards).

The Abstract section outlines clearly the problematic, aims, methodology and findings of the current study while reporting the main conclusions aroused. The Introduction section is well structured and aiming and underlines appropriately the whole subject under study. The aims of the study are also clear and understood. The Materials and methods section is clear, well written, and encloses all the information related to the adopted methodology, and statistical analysis. The Results section shows a correct statistical representation and an adequate scientific analysis of the findings. Similarly, the Discussion section compared the current study's findings with those of previously published studies in literature. Authors highlighted the main strength point of their study and its limitations; they further suggested related research being based on the raised assumptions and simulations.

My comments and queries for authors are detailed below in "Minor comments" section.

1.1. Minor comments:

1- The use of past verbal tense is needed when reporting the findings of the study.
2- The first voice form of the sentence shall be avoided and the impersonal form shall be adopted instead.
3- The references used in both Introduction and Discussion sections shall be up-to-date (2010 and onwards).
4- Abstract: Page 2, lines 27-30: "In this paper... layers": Kindly write the sentence in the past verbal tense form. Moreover, kindly avoid the first voice form of the sentence and adopt the impersonal form instead.
5- Abstract: Page 2, line 30: Kindly replace "is" by "was".
6- Abstract: Page 2, line 33: Kindly replace "are" by "were".
7- 1. Introduction: Page 2, lines 44-47: "These parameters... mesophyll": These statements lack reliable sources (references); accordingly, kindly provide them.
8- 1. Introduction: Page 3, lines 52-56: "In addition... mechanics": Both sentences repeat the same information; accordingly, kindly reformulate.
9- 1. Introduction: Page 3, lines 68-71: "For example... approaches": Kindly avoid the first voice form of the sentence and adopt the impersonal form instead.
10- 1. Introduction: Pages 3-4, lines 75-80: "To start... leaf": Same recommendation as in the previous comment.
11- 2. Materials and Methods, Plant material and growth conditions: Page 4, lines 86-87: Kindly mention the city and country of each research center.
12- 2. Materials and Methods, Plant material and growth conditions: Page 4, line 90: Kindly adjust as follow: "15 mL".
13- 2. Materials and Methods, Microscopy: Page 4, line 102: Kindly put a space between the number and the temperature's unit.
14- 2. Materials and Methods, Microscopy: Page 5, line 116: Kindly adjust as follow: "were taken".
15- 2. Materials and Methods, Computational modelling: Page 5, line 126: Kindly adjust as follow: "were designed".
16- 2. Materials and Methods, Computational modelling: Pages 5-6, lines 130-131 and 134: Kindly

replace "has" by "had".
17- 2. Materials and Methods, Computational modelling: Page 6, line 139: Kindly adjust as follow: "was tested".
18- 2. Materials and Methods, Computational modelling: Page 6, line 141: Kindly adjust as follow: "was simulated".
19- 2. Materials and Methods, Computational modelling: Page 6, line 144: Kindly adjust as follow: "were simulated".
20- 2. Materials and Methods, Computational modelling: Page 6, line 151: Kindly adjust as follow: "concentrations".
21- 3. Results: Page 7, lines 165-166: "To investigate... the leaf": Kindly avoid the first voice form of the sentence and adopt the impersonal form instead.
22- 3. Results: Page 7, lines 173-175: "We assigned... and shape": Same recommendation as in the previous comment.
23- 3. Results: Page 8, lines 183-186: "Rice... parameter": Same recommendation as in the previous two comments.
24- 3. Results: Page 8, lines 195-196: "To investigate... mesophyll": Same recommendation as in the previous comments.
25- 3. Results: Page 8, lines 196-201: "These data... 20-50{degree sign}": Kindly adopt the past verbal tense for these sentences.
26- 3. Results: Page 8, lines 202-206: "Having... O. meridionalis": Kindly avoid the first voice form of these sentences and adopt the impersonal form instead.
27- 3. Results: Page 9, line 214: Kindly replace "are" by "were".
28- 3. Results: Page 9, line 217: Kindly adjust as follow: "was calculated".
29- 3. Results: Page 9, line 218: Kindly replace "is" by "was".
30- 3. Results: Page 9, line 234: Kindly adjust as follow: "while investigating".
31- 3. Results: Page 10, lines 250-252: "To investigate... approach": Kindly avoid the first voice form of the sentence and adopt the impersonal form instead.
32- 3. Results: Pages 10-11, lines 253-266: Kindly adopt the past verbal tense for all these sentences.
33- 3. Results: Page 11, line 275: Kindly replace "has" by "had".
34- 4. Discussion, Rice mesophyll displays a conserved pattern of size and shape: Pages 11-12, lines 280-288: Kindly adopt the past verbal tense for all these sentences.
35- 4. Discussion, Rice mesophyll displays a conserved pattern of size and shape: Page 12, lines 288-290: "These observations... the leaf": The references used for this statement are very old; accordingly, kindly replace them by more recent ones (2010 and onwards).
36- 4. Discussion, Rice mesophyll displays a conserved pattern of size and shape: Page 12, line 302: Same recommendation as in the previous comment regarding the first reference.
37- 4. Discussion, Rice mesophyll displays a conserved pattern of size and shape: Page 13, line 325: "To make... approach": Kindly avoid the first voice form of the sentence and adopt the impersonal form instead.
38- 4. Discussion, Rice mesophyll displays a conserved pattern of size and shape: Page 13, line 327: Kindly adjust as follow: "led".
39- 4. Discussion, Rice mesophyll displays a conserved pattern of size and shape: Page 13, lines 334-335: "At present... speculate": Kindly remove this sentence.
40- 4. Discussion, Rice mesophyll displays a conserved pattern of size and shape: Page 13, line 337: Kindly replace "are" by "were".

Reviewer #2:

In this study, Jen et al. revealed that the size and shape of mesophyll cells are different depending on the layers in rice leaves, which are traditionally viewed as displaying a relatively uniform pattern. This pattern is confirmed by conducting a quantitative analysis based on microscopy images and computational modeling in the leaves of different rice cultivars. Notably, the author suggested that the significance of this pattern could be related to photosynthetic activities. I believe this study has a novelty and might help determine photosynthetic efficiency in grass plants. Overall, the article was well-written, and the experiments were well-designed. I could not find a significant mistake, and this manuscript could be accepted as it is.

DECISION LETTER- ROUND 2

November 1, 2023
Dr. Jen Sloan
University of Sheffield
Sheffield, N/A
United Kingdom

MSID: 2023-01290R1
MS TITLE: Conserved cellular patterning in the mesophyll of rice leaves

Dear Dr. Jen Sloan:

I am pleased to inform you that your manuscript "Conserved cellular patterning in the mesophyll of rice leaves" has been accepted for publication in Plant Direct.

Your article will appear online in the next available issue of Plant Direct. To ensure your article gets published as quickly as possible, please pay attention to the steps detailed below. We have found that most of the delays happen at this stage, especially at the payment stage, so please respond as quickly as possible when prompted.

License Agreement: Once your article has been accepted it will move to Production and undergo admin and file checking - you may receive an email with any queries we have at this stage. When all required items are received by the publisher and queries resolved, the corresponding author will receive an email from Wiley's Author Services system which will ask them to log on at https://authorservices.wiley.com/bauthor and will present them with the appropriate license for completion. Your article cannot be published until both the signed license agreement and payment of the article fee have been received.

Payment of the Open Access Article Publication Fee: All articles published in Plant Direct are fully open access: immediately and freely available to read, download and share. Plant Direct charges a publication fee to cover the publication costs. The corresponding author for this manuscript should have already received a quote from the payments team (cs-openaccess@wiley.com) with the estimated article publication fee; please email cs-openaccess@wiley.com if this has not been received. The corresponding author should log on to the Wiley Author Services site, where the

publication fee can be paid by credit card or an invoice. Pro Forma can also be requested. Payment of the publication charge must be received before the article will be published online.

Proofs: You will have the opportunity to look over your paper once more when you receive the author proofs for your article. The proofs will be with you in approximately two weeks. Please note that, in addition to publishing reviewer comments, the author's responses to review comments will also be published alongside the final version of the paper. If you would not like the author's responses to be published, please contact the editorial office at plantdirect@wiley.com .

Promotion of your article: You can help your research get the attention it deserves! Check out Wiley's free Promotion Guide for best-practice recommendations for promoting your work at www.wileyauthors.com/eeo/guide. And learn more about Wiley Editing Services which offers professional video, design, and writing services to create shareable video abstracts, infographics, conference posters, lay summaries, and research news stories for your research at www.wileyauthors.com/eeo/promotion.

Thank you again for your contribution to Plant Direct. If you have any questions, feel free to contact the editorial office at plantdirect@wiley.com .

Sincerely,

Reggie Millwood

Gustavo MacIntosh

Editor, Plant Direct

--- Editor comments

--- Reviewer comments:

AUTHORS' RESPONSE

Reggie Millwood and Gustavo MacIntosh,

Thank you for your comments, and for those of the reviewers. I have detailed below the changes we have made in response to them.

Jen Sloan

--- Editor comments:
Please add the figure number for each figure in your figure file.
Done

Please change the reference to Supp. Fig. 3 to Supp. Fig. S3 (see line 207).

Done

Please change the reference to Supp. Fig. 4 to Supp. Fig. S4 (see line 233).

Done

-- Reviewer comments:

Reviewer #1:

Comments to the Author:

Title: Conserved cellular patterning in the mesophyll of rice leaves

Overview and general recommendation:
The manuscript deals with an important topic related to the conserved cellular patterning in the mesophyll of rice leaves. The manuscript technically sounds well and shows high novelty. It is well written and shows the need for minor linguistic adjustments. In this regard, the needed adjustments are highlighted in "Minor comments" section. The use of past verbal tense is needed when reporting the findings of the study. The first voice form of the sentence shall be avoided and the impersonal form shall be adopted instead. The references used in both Introduction and Discussion sections shall be up-to-date (2010 and onwards).
The Abstract section outlines clearly the problematic, aims, methodology and findings of the current study while reporting the main conclusions aroused. The Introduction section is well structured and aiming and underlines appropriately the whole subject under study. The aims of the study are also clear and understood. The Materials and methods section is clear, well written, and encloses all the information related to the adopted methodology, and statistical analysis. The Results section shows a correct statistical representation and an adequate scientific analysis of the findings. Similarly, the Discussion section compared the current study's findings with those of previously published studies in literature. Authors highlighted the main strength point of their study and its limitations; they further suggested related research being based on the raised assumptions and simulations.
My comments and queries for authors are detailed below in "Minor comments" section.

We thank Reviewer #1 for their kind comments, and for the care they have taken with their extremely helpful recommendations throughout the text. We have taken all of this on board, as detailed below.

1.1. Minor comments:

1- The use of past verbal tense is needed when reporting the findings of the study.
2- The first voice form of the sentence shall be avoided and the impersonal form shall be adopted instead.
3- The references used in both Introduction and Discussion sections shall be up-to-date (2010 and onwards).

Thank you for this suggestion. We have taken on this advice and added more up to date references in a number of places in the text. In some places, we have not removed the older references, as we felt that including them helped to show that the conclusions mentioned are long-held. However, if the editor would prefer to remove the older references, then this is not a problem. In addition to the suggested additions, we have included (Terashima *et al.*, 2016; Gotoh *et al.*, 2018; Holloway-Phillips, 2019) in line 52.

4- Abstract: Page 2, lines 27-30: "In this paper... layers": Kindly write the sentence in the past verbal tense form. Moreover, kindly avoid the first voice form of the sentence and adopt the impersonal form instead.

Done

5- Abstract: Page 2, line 30: Kindly replace "is" by "was".

Done

6- Abstract: Page 2, line 33: Kindly replace "are" by "were".

Done

7- 1. Introduction: Page 2, lines 44-47: "These parameters... mesophyll": These statements lack reliable sources (references); accordingly, kindly provide them.

Done – added (Baillie and Fleming, 2020; Theroux-Rancourt *et al.* 2021) in line 45-46 and (Baillie and Fleming, 2020) in line 48.

8- 1. Introduction: Page 3, lines 52-56: "In addition... mechanics": Both sentences repeat the same information; accordingly, kindly reformulate.

Deleted first sentence.

9- 1. Introduction: Page 3, lines 68-71: "For example... approaches": Kindly avoid the first voice form of the sentence and adopt the impersonal form instead.

Reworded as follows:

"For example, in recent work from the Fleming group, a computational model of rice leaf photosynthesis, *eLeaf* was developed, which incorporated a 3D structure of IR64 rice leaves abstracted from image data of rice leaves at various resolutions using a range of approaches (Xiao *et al.*, 2023)."

10- 1. Introduction: Pages 3-4, lines 75-80: "To start... leaf": Same recommendation as in the previous comment.

Reworded as follows:

"To start to address this issue, a quantitative analysis of the mesophyll was performed in both IR64 and a range of other rice cultivars and species. The results, reported here, indicate that the rice leaf mesophyll had a more complex and ordered cellular architecture than previously acknowledged. A 2D modelling approach was then utilised, and the significance of this particular mesophyll structure to the photosynthetic performance of the rice leaf was explored."

11- 2. Materials and Methods, Plant material and growth conditions: Page 4, lines 86-87: Kindly mention the city and country of each research center.

Added

12- 2. Materials and Methods, Plant material and growth conditions: Page 4, line 90: Kindly adjust as follow: "15 mL".

Done

13- 2. Materials and Methods, Microscopy: Page 4, line 102: Kindly put a space between the number and the temperature's unit.

Done

14- 2. Materials and Methods, Microscopy: Page 5, line 116: Kindly adjust as follow: "were taken".

Done

15- 2. Materials and Methods, Computational modelling: Page 5, line 126: Kindly adjust as follow: "were designed".

Done, also changed the word equals to equalled twice in the following sentence.

16- 2. Materials and Methods, Computational modelling: Pages 5-6, lines 130-131 and 134: Kindly replace "has" by "had".

Done

17- 2. Materials and Methods, Computational modelling: Page 6, line 139: Kindly adjust as follow: "was tested".

Done

18- 2. Materials and Methods, Computational modelling: Page 6, line 141: Kindly adjust as follow: "was simulated".

Done

19- 2. Materials and Methods, Computational modelling: Page 6, line 144: Kindly adjust as follow: "were simulated".

Done

20- 2. Materials and Methods, Computational modelling: Page 6, line 151: Kindly adjust as follow: "concentrations".

Done, also replaced 'are' with 'were' in line 155.

21- 3. Results: Page 7, lines 165-166: "To investigate... the leaf": Kindly avoid the first voice form of the sentence and adopt the impersonal form instead.

Reworded as follows:

"To investigate whether there was a pattern of mesophyll size and shape, mesophyll cells were assigned to different layers (1 to 5) within the leaf (Fig. 1A)."

22- 3. Results: Page 7, lines 173-175: "We assigned... and shape": Same recommendation as in the previous comment.

Reworded as follows:

"Mesophyll cells were assigned to the different cell layers, then the cells were analysed for a range of quantitative parameters linked to size and shape (Fig. 1B-F)."

23- 3. Results: Page 8, lines 183-186: "Rice... parameter": Same recommendation as in the previous two comments.

Reworded as follows:

"Rice mesophyll cells are characterised by being highly lobed (Sage and Sage 2009), therefore this trait was also quantified, using a calculation of the ratio of actual cell perimeter to the minimal energy circumference of each cell as a measure of this parameter."

24- 3. Results: Page 8, lines 195-196: "To investigate... mesophyll": Same recommendation as in the previous comments.

Reworded as follows:

"To investigate this, measurements were made of the angle of the long axis of individual mesophyll cells in each layer of the mesophyll (Fig. 1F)."

25- 3. Results: Page 8, lines 196-201: "These data... 20-50{degree sign}": Kindly adopt the past verbal tense for these sentences.

Reworded as follows:

"These data generally substantiated the impression obtained from Fig 1E, i.e., the Layer 3 cells had a long axis which was predominantly aligned with the horizontal axis, which was also true for Layer 2 and Layer 4 cells. This equates to the mediolateral plane of the rice leaf, at right angles to the long axis of the leaf. The sub-epidermal mesophyll cells in Layer 1 displayed a greater variation in cell axis orientation, and Layer 5 cells were mostly orientated between 20-50°."

26- 3. Results: Page 8, lines 202-206: "Having... O. meridionalis": Kindly avoid the first voice form of these sentences and adopt the impersonal form instead.

Reworded as follows:

"In order to establish whether this pattern of mesophyll cell size and shape in IR64 leaves was reflected in the wider rice family, the mesophyll was studied in a range of rice varieties, including three cultivated *Oryza sativa* Indica variants (MRQ76, MR220 and Malinja), and three wild species (*O. latifolia*, *O. punctata* and *O. meridionalis*)."

Also changed 'show' to 'showed' in line 201.

27- 3. Results: Page 9, line 214: Kindly replace "are" by "were".

Done

28- 3. Results: Page 9, line 217: Kindly adjust as follow: "was calculated".

Done

29- 3. Results: Page 9, line 218: Kindly replace "is" by "was".

Done

30- 3. Results: Page 9, line 234: Kindly adjust as follow: "while investigating".

Done

31- 3. Results: Page 10, lines 250-252: "To investigate... approach": Kindly avoid the first voice form of the sentence and adopt the impersonal form instead.

Reworded as follows:

"To investigate how the pattern of mesophyll cell size revealed by this analysis might influence leaf photosynthetic performance in terms of basic light absorption and carbon assimilation rate, an initial 2D modelling approach was utilised."

32- 3. Results: Pages 10-11, lines 253-266: Kindly adopt the past verbal tense for all these sentences.

Reworded as follows:

"Model 1 was most representative of mesophyll described in this study, with five layers, in which the middle layer (Layer 3) was characterised by having relatively large cells (Fig. 7A). Model 2 had the same number of layers as Model 1, but the cells were all uniform and relatively small (Fig. 7B).

Consequently, the Model 2 leaf was slightly thinner than Model 1. Models 3 and 4 were made up entirely of relatively large cells with four and five layers, respectively (Fig. 7C,D). Model 4 was therefore slightly thicker than Models 1, 2 and 3. Each cell was modelled to have a proportion of plastid inversely proportional to cell size and a proportion of cytosol proportional to cell size, reflecting the findings of Sage and Sage (2009). Cell wall thickness and mitochondrial size were the same in all models. As a consequence of these parameters and packing, Models 1, 2 and 4 had the same plastid and cytosol volumes (Fig. 7E), but differed in their leaf thickness. Model 3 had the lowest plastid and cytosol volume. The amount of cell wall in contact with the air (S_{mes}) was very similar in Models 1 and 2, lowest in Model 3 and intermediate in Model 4 (Fig. 7E)."

33- 3. Results: Page 11, line 275: Kindly replace "has" by "had".

Done

34- 4. Discussion, Rice mesophyll displays a conserved pattern of size and shape: Pages 11-12, lines 280-288: Kindly adopt the past verbal tense for all these sentences.

Reworded as follows:

"The analysis reported here indicated that rice leaves from a range of genotypes and variants display a conserved pattern of cellular architecture. In particular, mesophyll cells in the middle (Layer 3) of the leaves were larger than mesophyll cells in other layers (Fig. 1B, Fig. 3, Fig. 4A), had the lowest degree of circularity (Fig. 1C, Fig. 4B, Fig. 5) and tended to have a cell long axis oriented orthogonal to the longitudinal axis of the leaf, thus aligning with the medio-lateral plane of the leaf (Fig 1E, Fig. 6). A distinctive pattern of cell axiality was also observed in the most adaxial mesophyll layer (Layer 1) where cells displayed a much wider range of long axis orientation than cells in the other layers of the mesophyll. These cells were also most likely to be ranked as the least lobed (Fig. 1D, Fig. 4C)."

35- 4. Discussion, Rice mesophyll displays a conserved pattern of size and shape: Page 12, lines 288-290: "These observations... the leaf": The references used for this statement are very old; accordingly, kindly replace them by more recent ones (2010 and onwards).

Replaced with Beck, 2010, Pyke, 2012

Also replaced 'Our data' with 'The data presented in this article' in line 298 to avoid the first person voice.

36- 4. Discussion, Rice mesophyll displays a conserved pattern of size and shape: Page 12, line 302: Same recommendation as in the previous comment regarding the first reference.

This sentence is specifically referring to the long-established belief that monocot mesophyll is uniform, hence we have used older references as well as Beck, 2010 and Pyke, 2012.

Have also updated the reference in line 311 to Terashima *et al.*, 2016; Holloway-Phillips, 2019.

37- 4. Discussion, Rice mesophyll displays a conserved pattern of size and shape: Page 13, line 325: "To make... approach": Kindly avoid the first voice form of the sentence and adopt the impersonal form instead.

Reworded as follows:

"To make an initial analysis of this problem a modelling approach was taken."

38- 4. Discussion, Rice mesophyll displays a conserved pattern of size and shape: Page 13, line 327: Kindly adjust as follow: "led".

Done

39- 4. Discussion, Rice mesophyll displays a conserved pattern of size and shape: Page 13, lines 334-335: "At present... speculate": Kindly remove this sentence.

Removed

40- 4. Discussion, Rice mesophyll displays a conserved pattern of size and shape: Page 13, line 337: Kindly replace "are" by "were".

Done

Reviewer #2:

In this study, Jen et al. revealed that the size and shape of mesophyll cells are different depending on the layers in rice leaves, which are traditionally viewed as displaying a relatively uniform pattern. This pattern is confirmed by conducting a quantitative analysis based on microscopy images and computational modeling in the leaves of different rice cultivars. Notably, the author suggested that the significance of this pattern could be related to photosynthetic activities. I believe this study has a novelty and might help determine photosynthetic efficiency in grass plants. Overall, the article was well-written, and the experiments were well-designed. I could not find a significant mistake, and this manuscript could be accepted as it is.

We thank Reviewer #2 for their kind comments.
